# Asymmetric exponential amplification reaction on a toehold/biotin featured template: an ultrasensitive and specific strategy for isothermal microRNAs analysis

**DOI:** 10.1093/nar/gkw504

**Published:** 2016-06-02

**Authors:** Jun Chen, Xueqing Zhou, Yingjun Ma, Xiulian Lin, Zong Dai, Xiaoyong Zou

**Affiliations:** 1 School of Chemistry and Chemical Engineering, Sun Yat-Sen University, Guangzhou 510275, China; 2 College of Pharmacy, Guangdong Pharmaceutical University, Guangzhou 510006, China; 3 SYSU-CMU Shunde International Joint Research Institute, Shunde, Guangdong 528300, China

## Abstract

The sensitive and specific analysis of microRNAs (miRNAs) without using a thermal cycler instrument is significant and would greatly facilitate biological research and disease diagnostics. Although exponential amplification reaction (EXPAR) is the most attractive strategy for the isothermal analysis of miRNAs, its intrinsic limitations of detection efficiency and inevitable non-specific amplification critically restrict its use in analytical sensitivity and specificity. Here, we present a novel asymmetric EXPAR based on a new biotin/toehold featured template. A biotin tag was used to reduce the melting temperature of the primer/template duplex at the 5′ terminus of the template, and a toehold exchange structure acted as a filter to suppress the non-specific trigger of EXPAR. The asymmetric EXPAR exhibited great improvements in amplification efficiency and specificity as well as a dramatic extension of dynamic range. The limit of detection for the let-7a analysis was decreased to 6.02 copies (0.01 zmol), and the dynamic range was extended to 10 orders of magnitude. The strategy enabled the sensitive and accurate analysis of let-7a miRNA in human cancer tissues with clearly better precision than both standard EXPAR and RT-qPCR. Asymmetric EXPAR is expected to have an important impact on the development of simple and rapid molecular diagnostic applications for short oligonucleotides.

## INTRODUCTION

MicroRNAs (miRNAs), which are naturally occurring, highly conserved families of transcripts (1,2), are believed to be critical in the regulation of cell proliferation and metabolism (3), cell death (4), haematopoiesis (5), neuron development (6), human tumorigenesis (7), DNA methylation and chromatin modification (8). The sensitive and specific profiling of miRNAs is significant and fundamental for biological research and disease diagnostics (9) but is difficult due to the small size of miRNAs (18–25 nt in length), the sequence homology among family members, and their low abundance (10). The routine techniques for miRNA analysis, such as northern blotting and microarrays (11,12), either are insensitive or require expensive instruments. Polymerase chain reaction (PCR)-based methods are reliable and sensitive but require thermal cycling to achieve amplification. Homogeneous isothermal amplification assays, which benefit from usually faster binding processes in solution and an isothermal nature (13), are becoming increasingly important in facilitating the sensitive analysis of miRNAs.

**Figure 1.**
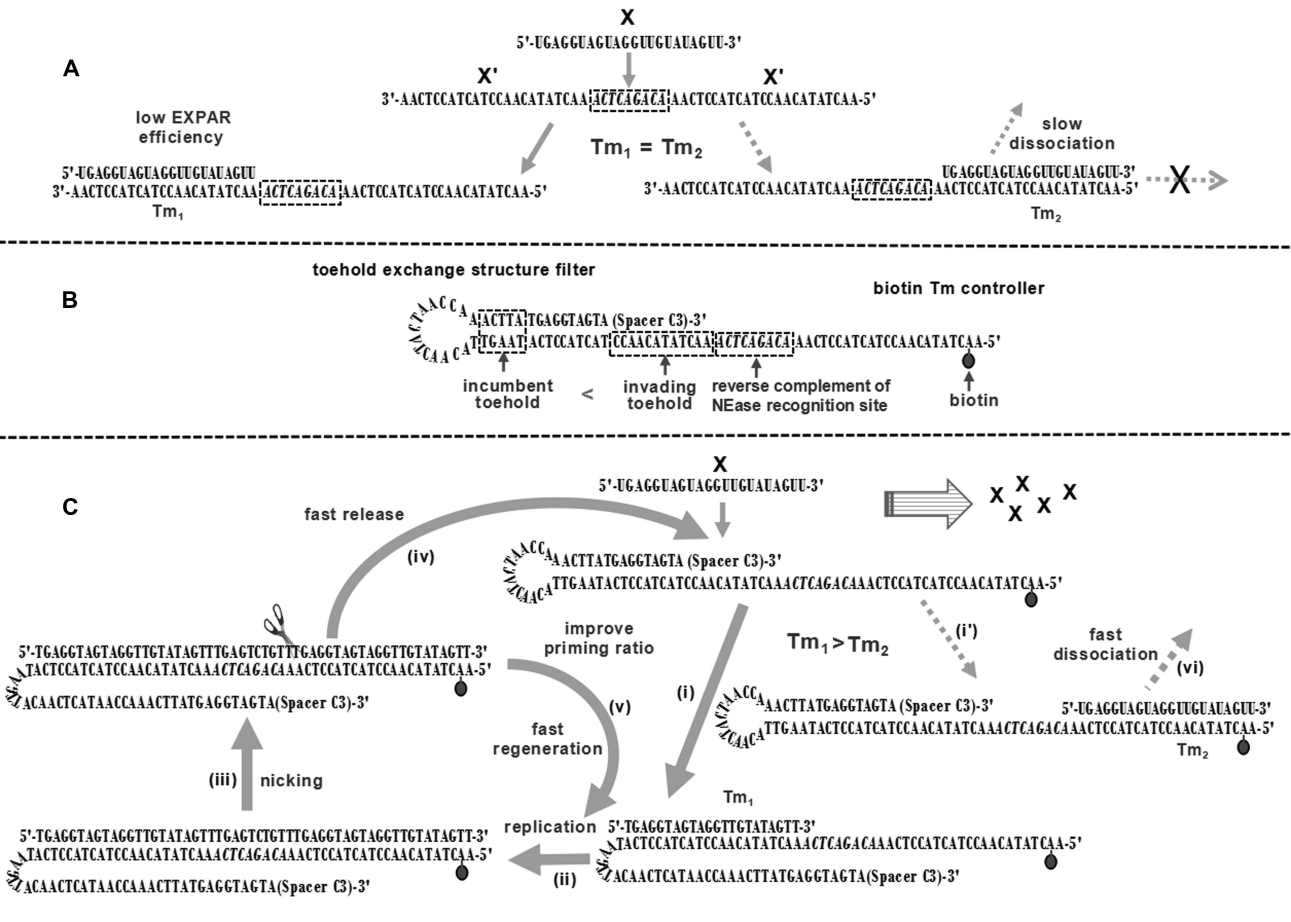
Mechanism of asymmetric exponential amplification. (**A**) General scheme for EXPAR on a standard template. (**B**) Structure of toehold/biotin-template-2. (**C**) General scheme for EXPAR on the toehold/biotin template. The reaction involves six steps: hybridization of the primer with (i) 3‘X'T or (i') 5‘X'T; (ii) polymerization of the primer from its 3′ terminus along the template by DNA polymerase; (iii) cleavage of the upper strand DNA by NEase; (iv) release of a copy of the primer from the template; (v) regeneration of the primer/template duplex and (vi) dissociation of the primer from 5‘X'T.

Isothermal exponential amplification reaction (EXPAR), as one of the most important nucleic acid amplification strategies, has been successfully applied in the analysis of DNAs (14,15), miRNAs (16–18), polymorphic sites in genomic DNA (19,20) and even proteins (21–23). Standard EXPAR is a circuiting-feedback process that combines polymerase strand extension and single strand nicking on a symmetrical template (24). Using let-7a miRNA as a model, a scheme for standard EXPAR is shown in Figure 1A. The standard template contains two copies of the target let-7a (X) reverse complement (X') at its 3′ and 5′ termini, separated by the reverse complement of the nicking endonuclease (NEase) recognition site and a required post-cut site spacer. The target let-7a forms a hybrid with the X' at the 3′ terminal region of the template (3‘X'T) and serves as a primer for a DNA polymerase to synthesize a complement of the template, thereby forming a double-stranded DNA (dsDNA) containing the NEase recognition site in the middle. NEase then nicks the upper strand and releases a copy of X that is able to prime other amplification templates. DNA polymerase elongates the recessed 3′-hydroxyl created by the departing X and repeats the process. The cycling of replication, nicking, and release reactions results in an exponential replication of target X with relatively high speed, good tolerance for inhibitory components in clinical samples, and excellent flexibility for the coupling of multiple reactions (13).

Despite impressive advantages, standard EXPAR is restricted by two bottlenecks. The first is a large loss of amplification efficiency. The prerequisite for the initiation of EXPAR is the formation of an extensible primer/template duplex, which requires a 3′-hydroxyl-terminated oligonucleotide to anneal with the proper configuration of the template (3′ terminus). However, the possible duplex formed by the primer with the X' at the 5′ terminus of the template (5‘X'T) is inextensible (Figure 1A). Although this transient duplex might dissociate and form an extensible primer/template duplex later (24), the extension reaction is likely to be delayed. The loss of extensible primer/template duplexes during each cycle greatly reduces the number of amplification events, prolongs the reaction time, and even causes failure of EXPAR for trace target oligonucleotides. The second disadvantage of standard EXPAR is the inevitable non-specific amplification. The amplification specificity of EXPAR is ensured by the Watson–Crick base pair (bp) interactions between primer and template. Double-stranded DNAs longer than 14 bp are insensitive to single nucleotide variations (25). Moreover, the DNA polymerization reaction is not highly sequence-restrictive and is likely to occur when nine consecutive base pairs are present at the 3′ terminus of the primer/template duplex (26). Therefore, when closely related miRNA family members anneal with 3‘X'T, replication and nicking reactions can still occur, producing a copy of the 'right' primer and triggering EXPAR. These limitations obviously restrict EXPAR in terms of analytical sensitivity, specificity, and speed. A breakthrough in these bottlenecks is highly demanded and envisioned to extend the practical scope of EXPAR.

Here, we present a novel asymmetric EXPAR based on a new amplification template. As shown in Figure 1B, the new amplification template is constructed from a standard template and features a biotin tag and a toehold exchange hairpin at its 5′ and 3′ termini, respectively (toehold/biotin template). The toehold structure has an invading strand that is six bases longer than the incumbent strand to maintain a high hybridization rate between the primer and template (27). The biotin tag is used to reduce the melting temperature (*T*_m_) of the primer/template duplex at the 5‘X'T, and the toehold exchange structure is used to suppress non-specific amplification. Therefore, target X has a higher chance to open the toehold hairpin and form more stable hybrid with the template (i) and is extended by DNA polymerase (ii). The nicking of the formed dsDNA (iii) produces a copy of X from the template (iv), which can initiate the next round of asymmetric EXPAR. The regenerated primer/template duplex (v) is ready for the next replication-nicking reaction (Figure 1C). As a result, the ratio of extensible primer/template duplexes formed during each cycle is improved, which greatly accelerates the entire amplification reaction by expediting the departure of the copy of the primer from the template (iv), the regeneration of the primer/template duplex (v), and the dissociation of the primer from 5‘X'T (vi). The proposed strategy shows clear improvements in sensitivity, specificity, and dynamic range over the standard methods and enables the sensitive and reliable analysis of let-7a miRNA in human cancer tissues with better precision than standard EXPAR and RT-qPCR.

## MATERIALS AND METHODS

### Materials

Oligonucleotides were obtained from TaKaRa Co. Ltd. (Dalian, China) and subsequently purified by high-performance liquid chromatography. Their sequences are listed in detail in Supplementary Table S1.

RNase inhibitor and DEPC-treated water were obtained from TaKaRa Biotechnology Co. Ltd. (Dalian, China; DEPC = diethylpyrocarbonate). Vent (exo-) DNA polymerase and Nt.BstNBI NEase were purchased from New England Biolabs (Beijing, China). Streptavidin (SA) and deoxyribonucleotide triphosphates (dNTPs) were purchased from Shanghai Sangon Bio. Eng. Tech. & Services Co. Ltd. (Shanghai, China). SYBR Green I (20 × stock solution in dimethyl sulfoxide, 20 μg ml^−1^) was purchased from Xiamen Bio-Vision Biotechnology (Xiamen, China). All of the solutions used in the EXPAR experiments were prepared with DEPC-treated deionized water.

### Analytical protocol

Two reaction solutions, A and B, were prepared on ice separately. Solution A consisted of Nt.BstNBI buffer (25 mM Tris–HCl, pH 7.9, 50 mM NaCl, 5 mM MgCl_2_, 0.5 mM dithiothreitol; Tris = 2-amino-2-hydroxymethylpropane-1,3-diol), SA, amplification template, dNTPs, RNase inhibitor and target miRNA. Solution B was composed of ThermoPol buffer (20 mM Tris–HCl, pH 8.8, 10 mM KCl, 10 mM (NH_4_)_2_SO_4_, 2 mM MgSO_4_, 0.1% Triton X-100), Nt.BstNBI NEase, Vent (exo-) DNA polymerase, and SYBR Green I. After preparation, solutions A and B were mixed immediately and adjusted with DEPC-treated deionized water to a final volume of 10 μl containing 0.1 mM amplification template, 0.2 mM SA, 250 mM dNTPs, 0.4 U ml^−1^ Nt.BstNBI, 0.05 U ml^−1^ Vent (exo-) DNA polymerase, 0.8 U ml^−1^ RNase inhibitor, 0.4 mg ml^−1^ SYBR Green I, 1× ThermoPol buffer, and 0.5 × Nt.BstNBI buffer. The mixture was placed in a StepOneplus Real-Time PCR System (Applied Biosystems, USA) and reacted at 55°C. The real-time fluorescence intensity was monitored using an isothermal protocol at 60 s intervals.

### Fluorescence measurements

Fluorescence emission spectra were obtained using a RF-5301PC fluorophotometer (Shimadzu, Japan). The emission spectra of FAM were obtained by exciting the samples at 490 nm and scanning the emission from 500 to 640 nm. The emission spectra of CY5 were obtained by exciting the samples at 640 nm and scanning the emission from 650 to 690 nm. All experiments were conducted in a final volume of 100 μl containing 10 nM fluorescent dye-labeled standard template or biotin template 2, 10 nM quencher-labeled let-7a, 0.2 mM SA, 1× ThermoPol buffer, and 0.5× Nt.BstNBI buffer at 55°C for 60 min. The fluorescence intensities were detected before and after hybridization with the quencher-labeled let-7a.

### RNA extraction

Human lung cancer tissues and cervical cancer tissues were obtained from Guangzhou Huayin Medical laboratory Center of Southern Medical University. Fresh tissues were added to 0.8 ml of trizol and vortexed vigorously to a homogenous lysate. The lysate was added to 0.2 ml of chloroform, vortexed for 15 s and then incubated at 25–30°C for 2–3 min. The resultant mixture was centrifuged at 4°C for 15 min at 12 000 rpm to separate the aqueous and organic phases. The aqueous phase was transferred into a fresh tube and added to an equal volume of isopropyl alcohol. After incubation at 15–30°C for 10 min and centrifugation at 4°C for 10 min at 12 000 rpm, the resultant aqueous phase was discarded, and the precipitate was washed with 800 μl of 75% alcohol. The resultant precipitate was further centrifuged at 4°C for 5 min at 7500 rpm. The residual alcohol and the obtained total RNA were eluted with DEPC-treated water. The integrity of total RNA was assessed by gel electrophoresis and stored at 80°C for further use.

### Reverse transcription

The reverse transcription of the obtained total RNA was carried out in a mixture containing 12.5 μl of RNase-free doubly distilled water, 2 μl of 5× PrimeScript™ Buffer (for real time), 1 μl of Prime Script™ RT Enzyme Mix I, 0.5 μl of let-7a RT Primer (10 pmol μl^−1^) and 4 μl of the extracted total RNA. The mixture was incubated at 37°C for 40 min followed by heat inactivation of reverse transcriptase at 85°C for 2 min. The obtained cDNA were store at −20°C for future use.

### Quantitative RT-qPCR analysis

Quantitative real-time fluorescence analysis was performed on a 7300 Real-Time PCR System (Applied Biosystems, USA) based on the protocol of literature (28). Forty cycles of amplification were performed. Each cycle comprised an initial denaturation step at 95°C for 2 min, a denaturation step at 93°C for 15 s, and annealing and extension steps at 55°C for 25 s. The detection system was 25 μl of a mixture containing 18.5 μl of doubly distilled water, 2.5 μl of 10× PCR buffer (with SYBR Green I and ROX), 0.5 μl of dNTPs, 0.5 μl of TAQ, 2 μl of cDNA, 0.5 μl of forward primer and 0.5 μl of reverse primer (Supplementary Table S1). The *Ct* values were converted into absolute let-7a copy numbers using a standard curve obtained from synthetic let-7a miRNA.

## RESULTS

### Mathematical model of EXPAR

**Figure 2.**
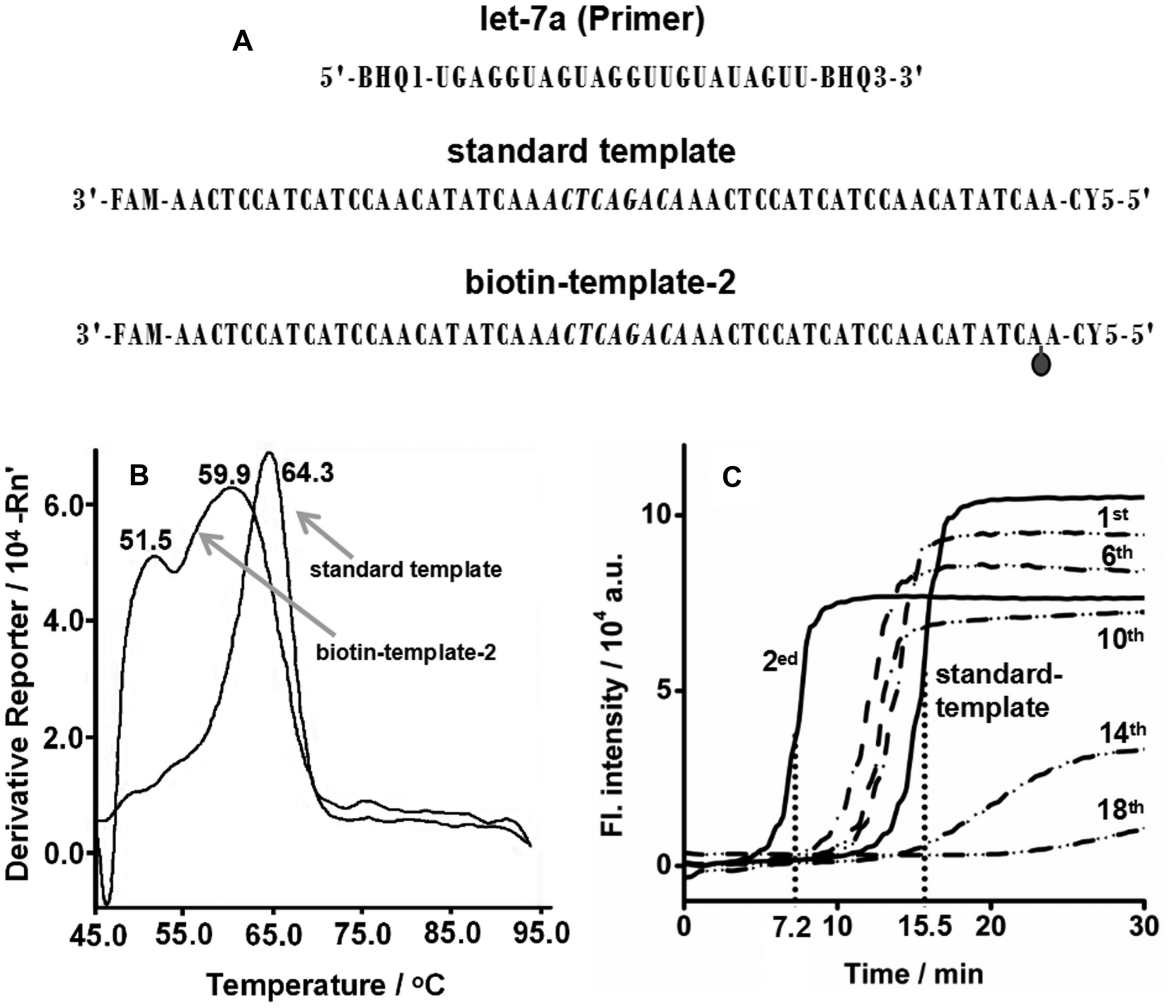
Effect of biotin tag on EXPAR. (**A**) Diagram of the experimental setup. (**B**) Determination of Tm of duplexes of let-7a/standard template and let-7a/biotin-template-2 by a StepOneplus Real-Time PCR System (Applied Biosystems, USA) using SYBR Green I as the reporter dye. The biotin-template-2 is a standard template labeled with a biotin at the second base from its 5′ terminus. *T*_m_ curves were obtained from the derivative of the fluorescence intensity as a function of temperature. The temperature was increased from 45°C to 95°C at a rate of 5°C min^–1^. (**C**) Real-time fluorescence curves of EXPAR reactions at 55°C with 0.05 U μl^–1^ Vent (exo-) DNA polymerase, 0.4 U μl^–1^ Nt.BstNBI NEase, 6.02 × 10^9^ copies (10 fmol) let-7a and 1 μM standard template or biotin-template-1, 2, 6, 10, 14, 18. The biotin-template-1, 2, 6, 10, 14, 18 are standard templates labeled with a biotin at the first, second, sixth, 10th, 14th, 18th base from their 5′ terminus, respectively.

In principle, the EXPAR strategy is suitable for the amplification of any short oligonucleotide. For the proof-of-concept study, let-7a miRNA was chosen as a target model. The analysis of let-7a requires an extremely high sensitivity and specificity because let-7a is usually expressed at a very low level and co-exists with highly similar concomitant miRNAs (29,30). To evaluate the hybridization efficiency of target oligonucleotide to the template of standard EXPAR, the hybridization of let-7a with a standard template was investigated. The standard template was labeled with the fluorescent dyes FAM and CY5 at its 3′ and 5′ termini, respectively. Meanwhile, let-7a was tagged with the quencher molecules BHQ1 and BHQ3 at its 5′ and 3′ termini, respectively (Figure 2A). The FAM/CY5-labeled standard template initially showed fluorescence peaks at 522 nm (FAM) and 670 nm (CY5) (Supplementary Figures S1 and S2). After incubation of the FAM/CY5-labeled standard template with the BHQ1/BHQ3-labeled let-7a at 55°C for 1 h, the fluorescence intensity of FAM decreased by 51% (Supplementary Figure S1), whereas that of CY5 decreased by 54% (Supplementary Figure S2). Considering the quenching efficiency of BHQ1 to FAM is similar to that of BHQ3 to CY5 (31,32), and the sequences of 3‘X'T and 5‘X'T are the same, the let-7a hybrid with 3‘X'T and 5‘X'T is supposed to form at close ratios. Supposing that *φ* (1 ≥ *φ* ≥ 0) is the ratio of the target oligonucleotide that anneals with 3‘X'T, only approximately half of the annealed let-7a can form extensible primer/template duplexes during each EXPAR cycle.

The drop of *φ* greatly affects the amplification efficiency of EXPAR, which can be inferred from the mathematical model of EXPAR (see details in Supplementary information, S2). Assuming that annealing is a bimolecular and single-step reaction, EXPAR can be regarded as a consecutive reaction:

\begin{equation*}\rm{X/5'X'T}\,\,\, {\overset{\theta}{\Leftrightarrow}}\,\,\, \rm{X + T}\,\, \overset{\alpha}{\rightarrow}\,\,\, \rm{X/3'X'T}\,\,\, \overset{\epsilon }{\rightarrow}\,\,\, \rm{dsDNA}\,\,\, \overset{\gamma }{\rightarrow} \rm{X}\end{equation*}

where *θ* and *α* are the annealing rates of the X hybrid with 5‘X'T or 3‘X'T, respectively, *ϵ* is the conversion rate from X/3‘X'T to dsDNA, *γ* is the productive rate of X from dsDNA; thus,

(1) \begin{equation*}{c_{\rm{X}}} = {{\rm{e}}^{\beta t}}{c_{{{\rm{X}}_0}}}\end{equation*}

Where

\begin{equation*}\beta = \left( {\frac{{\gamma {\lambda ^2}}}{{\varepsilon \left( {\lambda + 1} \right)}} - \frac{{1 - \varphi }}{{\varphi \left( {\lambda + 1} \right)}} - {\rm{\alpha }}} \right){c_{{{\rm{T}}_0}}}\end{equation*}

Equation (1) clearly indicates that the target oligonucleotide is replicated exponentially with an amplification factor (*F*) of e*^β^*^t^ and that the value of *F* is positively related to *γ* and *φ*. The impact of *φ* on *F* can be roughly assessed from the final outcome after EXPAR. Assuming that *θ, α, ϵ* and *γ* are constant during EXPAR, the final outcome from one target oligonucleotide after cycle n is derived to be (*φ* + 1)^*n*^ (Supplementary Table S2). The ideal EXPAR, in which all primers form extensible primer/template duplexes during each cycle (*φ* = 1), produces a final outcome of 2^*n*^. Standard EXPAR only has an *φ* of ∼50%, resulting in a final outcome of 1.5^*n*^. The amplification factor seriously decreases with the cycle (Supplementary Figure S2), which requires a facile way to improve the ratio *φ* to enhance extensible primer/template duplex formation in EXPAR.

### EXPAR on a biotin template

The main strategy for improving *φ* is to differentiate the Tm of the primer/3‘X'T duplex from that of the primer/5‘X'T duplex. It is known that the *T*_m_ of a duplex depends on the tag on the DNA strand in the duplex (33). Therefore, an internal biotin tag was used to decrease the *T*_m_ of the hybrid formed between the template and target sequence. The biotin-template-2 has the same sequence and fluorophore labels as the standard template, except for a biotin tag at the second base from its 5′ terminus (Figure 2A). After incubation with SA, the FAM/CY5-labeled biotin-template-2 showed two fluorescence peaks that were identical to those from the FAM/CY5-labeled standard template (Supplementary Figures S1 and S2), which suggested that the tag of biotin-SA on the template does not affect the fluorescence signal level of the FAM and CY5 labels. After incubating the FAM and CY5-labeled biotin-template-2 with the BHQ1/BHQ3-labeled let-7a at 55°C for 1 h, the fluorescence intensity of FAM decreased by 93.1% (Supplementary Figure S1), whereas that of CY5 only decreased by 36.8% (Supplementary Figure S2). Apparently, more let-7a hybrid is formed with the 3‘X'T of the FAM/CY5-labeled biotin-template-2. The improvement of the hybridization ratio resulted from the decrease of the *T*_m_ of the let-7a/5‘X'T duplex, as shown in Figure 2B. The let-7a/standard template duplex showed a unique *T*_m_ of 64.3°C, thus confirming that the hybridization of let-7a with 3‘X'T or 5‘X'T has the same thermodynamics. In contrast, the let-7a/biotin-template-2 duplex showed Tm values of 59.9°C and 51.5°C. The new Tm corresponded to the let-7a/5‘X'T duplex. An approximately 10°C decrease in the *T*_m_ of the let-7a/5‘X'T duplex allowed the increased formation of the let-7a/3‘X'T duplex. At a temperature between the Tm values, the hybridization of let-7a with 3‘X'T is preferred, but the formation of let-7a/5‘X'T is suppressed. Consequently, the ratio *φ* is improved, and greater formation of extensible primer/template duplexes is achieved.

To further investigate the effect of the biotin tag, EXPAR was performed on a series of biotin templates and monitored by real-time fluorescence using SYBR Green I as the reporter dye. Templates labeled with biotin at the first, second, sixth, 10th, 14th and 18th bases from their 5′ terminus are denoted as biotin-template-1, 2, 6, 10, 14 and 18, respectively. As shown in Figure 2C, EXPAR experiments with 6.02 × 10^9^ copies (10 fmol) of let-7a produced sigmoidal curves with different points of inflection (POI, defined as the time corresponding to the maximum slope in the sigmoidal curve). EXPAR on a standard template gave a POI value of 15.5 min, whereas on biotin-template-2, 6, and 10, the POI values were decreased to 7.19, 12.0 and 12.7 min, respectively. The lower values of the POI indicate a significant acceleration of the EXPAR reaction, which was greater when the template was labeled with a biotin tag closer to its 5′ terminus.

It should be noted that EXPAR on biotin-template-1 showed only a slight improvement in POI (Figure 2C). Templates that were labeled with a biotin tag far away from its 5′ terminus (biotin-template-14 and 18) or with multiple biotin molecules (biotin-template-2, 6) did not promote the EXPAR reaction (Figure 2C and Supplementary Figure S3). This may be because template strands that are labeled with biotin far from its 5′ terminus or with multiple biotin molecules are likely to form a more looped structure in the presence of SA (34,35).

**Figure 3.**
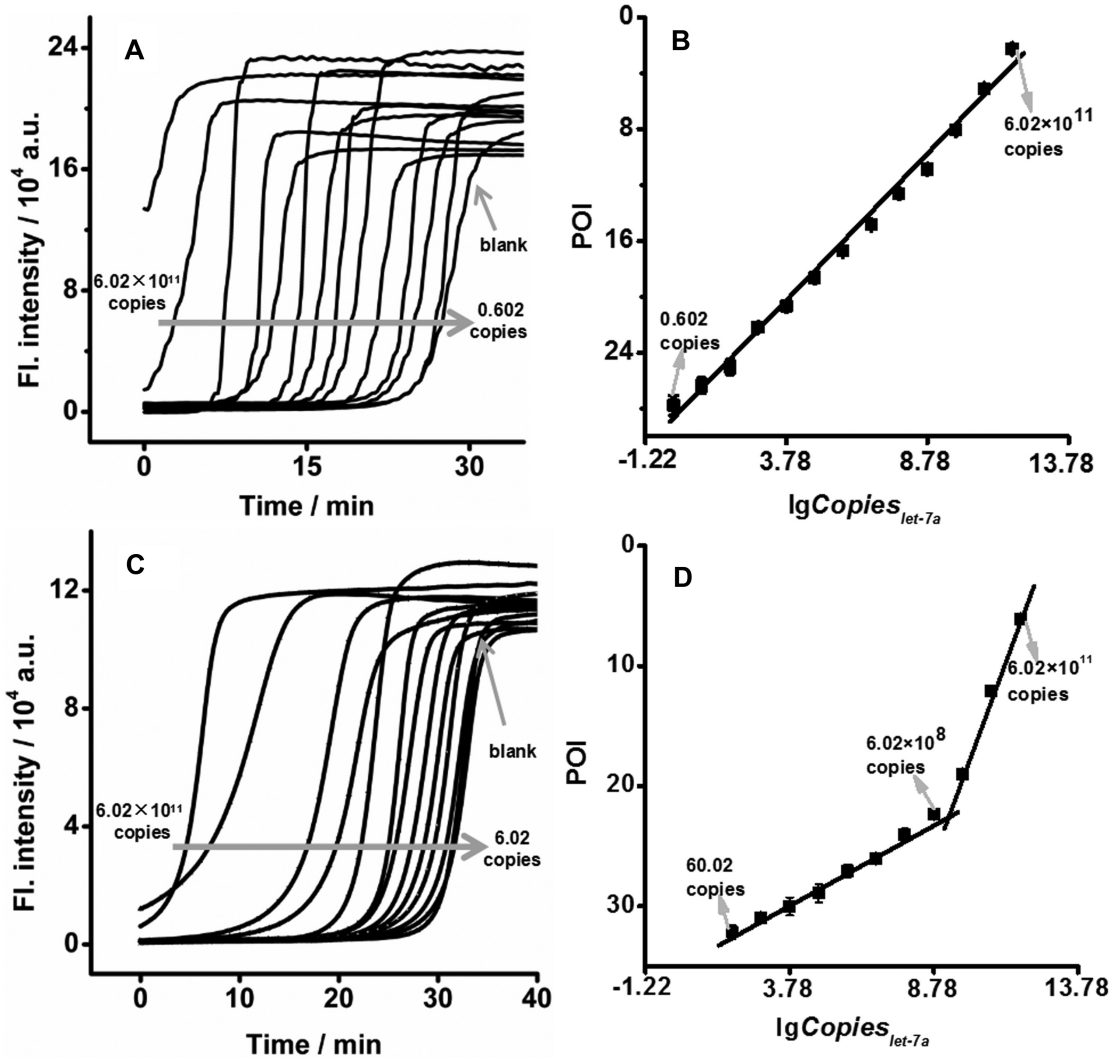
Comparison of standard EXPAR and biotin-based EXPAR. Real-time fluorescence curves of EXPAR on biotin-template-2 (**A**) or standard template (**C**) in response to different amounts of let-7a at 55°C. The biotin-template-2 is a standard template labeled with a biotin at the second base from its 5′ terminus. Plots for the POI of EXPAR on the biotin template (**B**) or standard template (**D**) versus logarithmic amount of let-7a.

The successful enhancement of *φ* using the biotin template is expected to improve the analytical performance of EXPAR. Under optimal conditions (Supplementary Figures S4–S7), the EXPAR on biotin-template-2 showed clearly differentiable sigmoidal curves in response to different amounts of let-7a (Figure 3A). In a logarithmic scale, the POI values decreased linearly with an increase in the amount of let-7a between 0.602 copies (0.001 zmol) and 6.02 × 10^11^ copies (100 fmol) (Figure 3B). The correlation equation was POI = 28.3 – 2.09lg*Copies*_let-7a_ (the correlation coefficient *R* was 0.996). In comparison, the responses of the standard EXPAR to these amounts of let-7a were all slower than those of biotin-template-based EXPAR (Figure 3C). Two linear correlations were obtained with narrower dynamic ranges from 60.02 copies (0.1 zmol) to 6.02 × 10^8^ copies (1.0 fmol) and from 6.02 × 10^8^ copies (1.0 fmol) to 6.02 × 10^11^ copies (1.0 pmol) (Figure 3D). The correlation equations were POI = 35.1 – 1.39lg*Copies*_let-7a_ (*R* = 0.994) and POI = 72.2 – 5.51lg*Copies*_let-7a_ (*R* = 0.992), respectively. The EXPAR on the biotin template successfully improved the limit of detection and dynamic range by 2 and 4 orders of magnitude, respectively, compared with the standard EXPAR.

**Figure 4.**
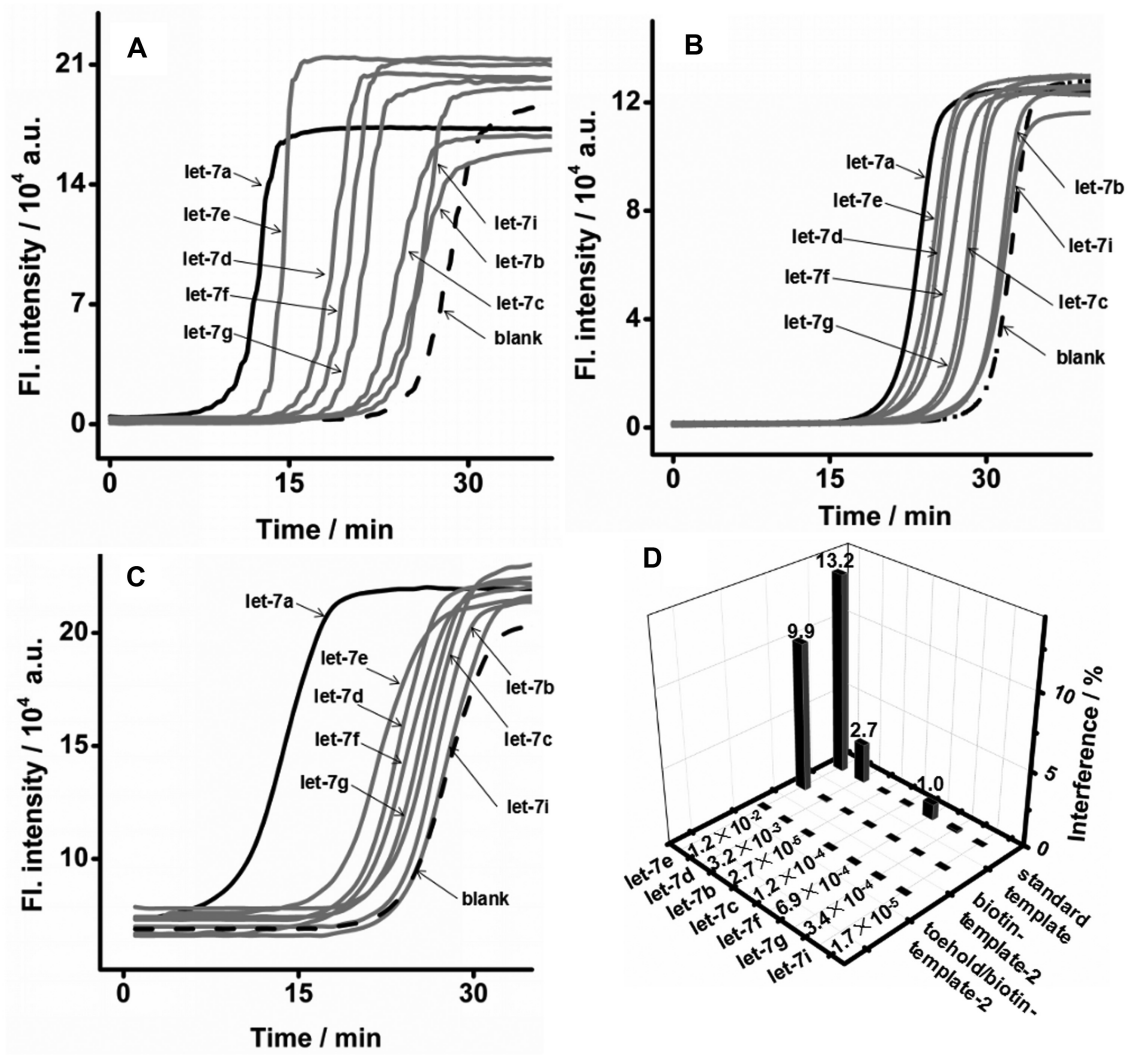
Investigation of the specificity of different EXPAR templates. Real-time fluorescence curves of EXPAR on (**A**) standard template, (**B**) biotin-template-2 or (**C**) toehold/biotin-template-2 in response to 6.02 × 10^9^ copies (10 fmol) let-7a, let-7b, let-7c, let-7d, let-7e, let-7f, let-7g and let-7i at 55°C. The biotin-template-2 is a standard template labeled with a biotin at the second base from its 5′ terminus. The toehold/biotin-template-2 is a standard template with a biotin at the second base from its 5′ terminus and a dumbbell-shaped hairpin at its 3′ terminus. (**D**) Histogram of the interferences to let-7a detection using standard EXPAR, biotin-template-based EXPAR and toehold/biotin-template-based EXPAR.

In addition to the ultrahigh sensitivity and wide dynamic range, EXPAR on biotin template also showed a clear improvement in amplification specificity for distinguishing concomitant miRNAs. The interference on the detection of let-7a from its family members was evaluated (Figure 4A and B, Supplementary Tables S3 and S4). In standard EXPAR, let-7b, let-7c, let-7g and let-7i showed negligible interference (<1%), whereas the interferences from let-7d (2.7%), let-7e (13.2%), and let-7f (1.04%) were more significant. In the case of EXPAR on biotin-template-2, in addition to a reduction of the interference from let-7b, let-7c, let-7 g and let-7i by approximately 1∼2 orders of magnitude, the interferences from let-7d, let-7e and let-7f were suppressed down to 0.044%, 9.93% and 0.023%, respectively. Biotin-template-based EXPAR is obviously better for discriminating all of the let-7 miRNA family members.

### EXPAR on a toehold/biotin template

Although the specificity of EXPAR is greatly improved using a biotin template, the interference of let-7e (9.93%) is still unacceptable if let-7a co-exists with a high concentration of let-7e. The development of a template that improves the specificity of EXPAR for let-7e without losing sensitivity is highly desired. Toehold exchange probes are capable of boosting the discrimination of robust sequences across complex environments by a factor of nearly a hundred times those of Watson–Crick base pairing or hairpin probes (27,36). Therefore, we further introduced a dumbbell-shaped hairpin at the 3′ terminus of biotin-template-2 to suppress non-specific amplification. The interference on the detection of let-7a from its family members was evaluated (Figure 4C and Supplementary Tables S5). As seen, interference from the signals of let-7 miRNA family members was significantly decreased (Figure 4D). The interfering signals of let-7c, let-7d, let-7e, let-7f and let-7g were 2 ∼ 4 orders of magnitude lower than those from the standard EXPAR. In particular, the interference from the signal of let-7e was reduced from 13.2% to 0.012% (Supplementary Tables S5). The hairpin structure of the template is stable and can be opened only by the perfectly matched primer due to its high *T*_m_ (66.61°C, Supplementary Figure S8). The toehold/biotin-template-based EXPAR has a sufficiently high specificity to resolve miRNAs with a single nucleotide variation.

**Figure 5.**
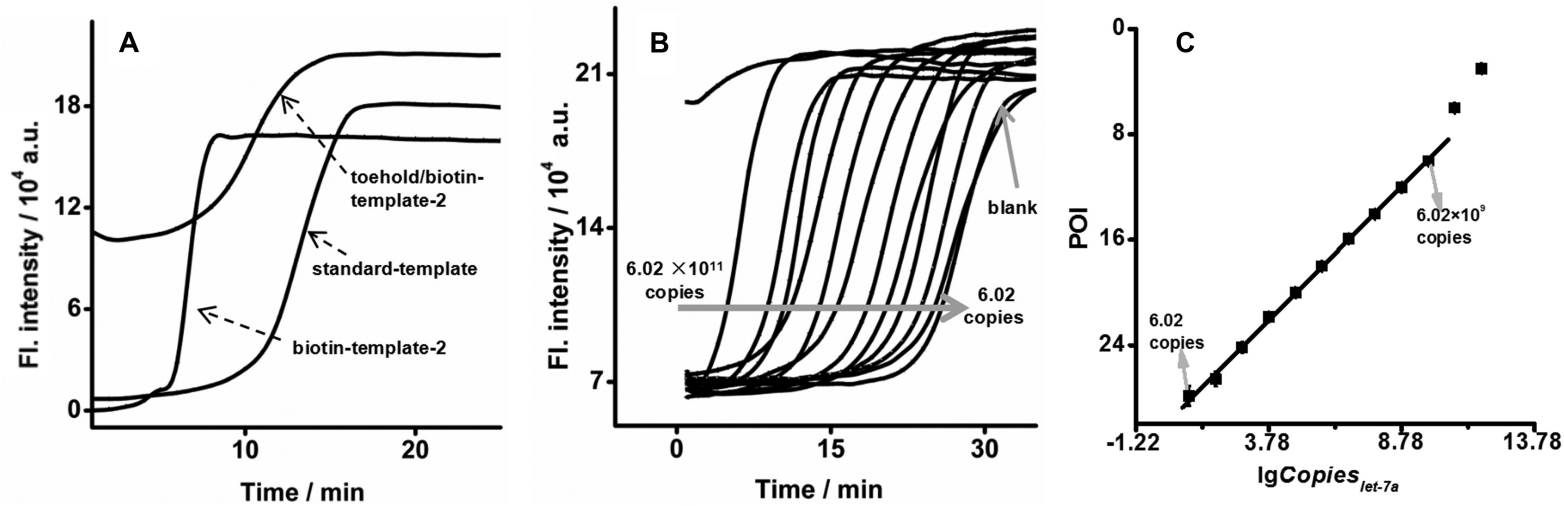
EXPAR on toehold/biotin-template-2. (**A**) Real-time fluorescence curves of EXPAR on standard template, biotin-template-2, or toehold/biotin-template-2 in response to 6.02 × 10^9^ copies (10 fmol) let-7a at 55°C. The biotin-template-2 is a standard template labeled with a biotin at the second base from its 5′ terminus. The toehold/biotin-template-2 is a standard template with a biotin at the second base from its 5′ terminus and a dumbbell-shaped hairpin at its 3′ terminus. (**B**) Real-time fluorescence curves of EXPAR on toehold/biotin-template-2 in response to different amounts of let-7a at 55°C. (**C**) Plots for the POI of EXPAR on toehold/biotin-template-2 versus logarithmic amount of let-7a.

In addition to the dramatic improvement in specificity, EXPAR on a toehold/biotin template exhibited a greatly enhanced dynamic range and sensitivity due to the high hybridization rate between the primer and template. As shown in Figure 5A, in response to 6.02 × 10^9^ copies (10 fmol) let-7a, the reaction speed of EXPAR on the toehold/biotin-template-2 was slightly slower than that of biotin-template-based EXPAR, but still faster than standard EXPAR. Under optimal conditions, let-7a was analysed using EXPAR on toehold/biotin-template-2 (Figure 5B). On a logarithmic scale, the POI value was linearly correlated with the amount of let-7a over a range from 6.02 copies (0.01 zmol) to 6.02 × 10^9^ copies (10 fmol) (Figure 5C). The correlation equation was POI = −29.7 + 1.99lg*Copies*_let-7a_ (*R* = 0.999).

### Practical feasibility

It is known that the expression level of let-7a is frequently related to non-small cell lung cancer (NSCLC) and cervical cancer (37–39). Therefore, these methods were applied to determine let-7a expression in total RNA samples from three human lung cancer tissues and two cervical cancer tissues. The let-7a level in the same RNA samples was also measured using standard RT-qPCR method (reference). As shown in Table 1, the results from biotin-template-based EXPAR and toehold/biotin-template-based EXPAR were consistent with those from the standard RT-qPCR method, with relative errors (RE) of <15%. Furthermore, the overall relative standard deviations (RSD) of the detection by biotin-template-based EXPAR and toehold/biotin-template-based EXPAR were smaller than those of RT-qPCR. The results suggest that the proposed method can accurately detect the expression of let-7a in human cancer samples with better precision than the standard method, thus holding a great potential for applications in clinical diagnosis.

**Table 1.** Levels of let-7a (×10^6^ copies μl^–1^) in RNA samples from lung cancer and cervical cancer tissues quantified by RT-qPCR, biotin-template-based EXPAR and toehold/biotin-template-based EXPAR

	RT-qPCR		EXPAR on biotin-template-2			EXPAR on toehold/biotin-template-2		
	*A*_let-7a_	SD (%)	*A*_let-7a_	SD (%)	RE (%)	*A*_let-7a_	SD (%)	RE (%)
Lung cancer 1	4.05	2.42	4.33	1.69	6.91	4.17	0.89	2.96
Lung cancer 2	0.418	25.6	0.373	23.3	-10.8	0.361	27.1	-13.6
Lung cancer 3	3.98	2.54	4.33	1.76	8.79	4.09	2.03	2.76
Cervical cancer 1	0.406	48.5	0.385	31.2	5.17	0.413	12.3	1.72
Cervical cancer 2	4.30	3.56	4.09	1.83	-4.88	4.87	1.62	13.3

## DISCUSSION

By running an asymmetric EXPAR on a toehold/biotin template, the bottlenecks of standard EXPAR, in terms of amplification efficiency and non-specific amplification, have been efficiently surmounted. The new EXPAR has all the major advantages of standard EXPAR. For example, its isothermal nature allows a wide applicability because neither a thermally stable enzyme nor sophisticated instrumentation is required to conduct the assay. Its homogeneous format is attractive for conducting a single-tube assay that minimizes the number of contamination-prone steps and simplifies assay procedures by eliminating separation or washing processes. It can be used in wider applications for different detection purposes because the signal amplification reaction can be easily triggered and multiplexed to obtain various signal outputs of colour, fluorescence or current (40–42). Moreover, the method provides the following outstanding features over standard EXPAR.

- Ultrahigh sensitivity and specificity with dramatically wide dynamic range. Pursuing higher sensitivity usually leads to a compromise in the dynamic range (43,44). However, the proposed methods not only achieved limits of detection for oligonucleotides down to 0.602 copies (0.001 zmol) and 6.02 copies (0.01 zmol), which are the most sensitive methods compared to the common nucleic acid amplification strategies (Supplementary Table S6), but also extended the dynamic range to 10–12 orders of magnitude. The method is envisioned to be more suitable for accurate and facile determination of a wide range of oligonucleotides in complex matrices under constant conditions.
- Simplicity and ease of adaptability. The presented method achieved great improvements by simply adding a toehold structure and a biotin tag to the template without any other reagents or complex structures. This means that the method, without additional complexity, can not only fulfil all the applications of standard EXPAR with better performance but also extend EXPAR into numerous applications that standard EXPAR cannot reach.

We believe that the method has great potential for application in biological research, diseases diagnostics, environmental analysis, food security and other areas.

## Supplementary Material

SUPPLEMENTARY DATA
