# Supplementary material for: Asymmetric exponential amplification reaction on a toehold/biotin featured template: an ultrasensitive and specific strategy for isothermal microRNAs analysis
Source: Nucleic Acids Res. 2016 Jun 2;44(15):e130. doi: 10.1093/nar/gkw504 (PMC5009742; doi:10.1093/nar/gkw504)
Supplement: SUPPLEMENTARY DATA [file supp_44_15_e130__index.html]

Asymmetric exponential amplification reaction on a toehold/biotin featured template: an ultrasensitive and specific strategy for isothermal microRNAs analysis — Asymmetric exponential amplification reaction on a toehold/biotin featured template: an ultrasensitive and specific strategy for isothermal microRNAs analysis — SUPPLEMENTARY DATA 

# Asymmetric exponential amplification reaction on a toehold/biotin featured template: an ultrasensitive and specific strategy for isothermal microRNAs analysis

## SUPPLEMENTARY DATA

- SUPPLEMENTARY DATA
