# Supplementary material for: Asymmetric exponential amplification reaction on a toehold/biotin featured template: an ultrasensitive and specific strategy for isothermal microRNAs analysis
Source: Nucleic Acids Res. 2016 Jun 2;44(15):e130. doi: 10.1093/nar/gkw504 (PMC5009742; doi:10.1093/nar/gkw504)
Supplement: SUPPLEMENTARY DATA [file supp_gkw504_nar-00483-met-g-2016-File004.pdf]

Supplementary Information

Asymmetric exponential amplification reaction on a toehold/biotin featured template: an ultrasensitive and specific strategy for isothermal microRNAs analysis

Jun Chen[1], Xueqing Zhou[1], Yingjun Ma[1], Xiulian Lin[2], Zong Dai[1,]* and Xiaoyong Zou[1,3]

---

1 School of Chemistry and Chemical Engineering, Sun Yat-Sen University

135 Xingang West Road, Guangzhou, China 510275

E-mail: daizong@mail.sysu.edu.cn

2 College of Pharmacy, Guangdong Pharmaceutical University, Guangzhou 510006, P. R. China

3 SYSU-CMU Shunde International Joint Research Institute, Shunde, Guangdong 528300, P. R. China

Contents

S1. Sequences of the used oligonucleotides..S3

S2. Mathematical model of EXPAR...S4

S3. Amplification fold simulation..S6

S4. Hybridization of primer with strandard or biotin template..S6

S5. Ideal and experimental amplification fold...S7

S6. EXPAR with biotin-template-2,6...S7

S7. Optimization of the amount of DNA polymerase..S8

S8. Optimization of the amount of NEase...S9

S9. Optimization of the amount of template...S10

S10. Optimization of reaction temperature...S11

S11. Estimation of interference of EXPAR.......... ....S11

S12. Melting temperature investigation of toehold/biotin-template-2....................................S13

S13. Comparison of the analytical performance of different methods...................................S13

S1. Table S1. Sequences of the used oligos[a]

Name	Sequence
let-7a	5'-UGAGGUAGUAGGUUGUAUAGUU-3'
let-7b	5'-UGAGGUAGUAGGUUGUGUGGUU-3'
let-7c	5'-UGAGGUAGUAGGUUGUAUGGUU-3'
let-7d	5'-AGAGGUAGUAGGUUGCAUAGU-3'
let-7e	5'-UGAGGUAGGAGGUUGUAUAGU-3'
let-7f	5'-UGAGGUAGUAGAUUGUAUAGUU-3'
let-7g	5'-UGAGGUAGUAGUUUGUACAGU-3'
let-7i	5'-UGAGGUAGUAGUUUGUGCUGUU-3'
BHQ1/BHQ3-labelled let-7a	5'-BHQ1-UGAGGUAGUAGGUUGUAUAGUU-BHQ3-3'
Standard template	5'-AACTATACAACCTACTACCTCAA*ACAGACTCA*AACTATACAACCTACTACCTCAA-3'
FAM/CY5-labelled standard template	5'-CY5-AACTATACAACCTACTACCTCAA*ACAGACTCA*AACTATACAACCTACTACCTCAA-FAM-3'
Biotin-template-1	5'-A(Biotin)ACTATACAACCTACTACCTCAA*ACAGACTCA*AACTATACAACCTACTACCTCAA-3'
Biotin-template-2	5'-AA(Biotin)CTATACAACCTACTACCTCAA*ACAGACTCA*AACTATACAACCTACTACCTCAA-3'
Biotin-template-6	5'-AACTAT(Biotin)ACAACCTACTACCTCAA*ACAGACTCA*AACTATACAACCTACTACCTCAA-3'
Biotin-template-10	5'-AACTATACAA(Biotin)CCTACTACCTCAA*ACAGACTCA*AACTATACAACCTACTACCTCAA-3'
Biotin-template-14	5'-AACTATACAACCTA(Biotin)CTACCTCAA*ACAGACTCA*AACTATACAACCTACTACCTCAA-3'
Biotin-template-18	5'-AACTATACAACCTACTAC(Biotin)CTCAA*ACAGACTCA*AACTATACAACCTACTACCTCAA-3'
Biotin-template-2,6	5'-AA(Biotin)CTAT(Biotin)ACAACCTACTACCTCAA*ACAGACTCA*AACTATACAACCTACTACCTCAA-3'
FAM/CY5-labelled biotin-template-2	5'-CY5-AA(Biotin)CTATACAACCTACTACCTCAA*ACAGACTCA*AACTATACAACCTACTACCTCAA-FAM-3'
Toehold/biotin-template-2	5'-AA(Biotin)CTATACAACCTACTACCTCAA*ACAGACTCA*AACTATACAACCTACTACCTCATAAGTTACAACTCATAACCAAACTTATGAGGTAGTA-3'
Forward primer	5'-CGTCGTGAGGTAGTAGGTTG-3'
Reverse primer	5'-GTGCAGGGTCCGAGG-3'
RT primer	5'-GTCGTATCCAGTGCAGGGTCCGAGGTATTCGCACTGGATACGACAACTAT-3'

[a] The underlined region identifies the complement of Nt.BstNBI recognition site.

S2. Mathematical model of EXPAR

Assuming that annealing is a bimolecular and single-step reaction, the EXPAR can be regarded as a consecutive reaction:

$$X/5'X'T \overset{\theta}{\Leftrightarrow} X+T \overset{\alpha}{\rightarrow} X/3'X'T \overset{\varepsilon}{\rightarrow} dsDNA \overset{\gamma}{\rightarrow} X$$

The consevutive reaction composes the following four reactions.

(I) $X + T \overset{\theta}{\Leftrightarrow} X/5'X'T$ (II) $X + T \overset{\alpha}{\rightarrow} X/3'X'T$

(III) $X/3'XT \overset{\varepsilon}{\rightarrow} dsDNA$ (IV) $dsDNA \overset{\gamma}{\rightarrow} X$

Where $\boldsymbol{\theta}$ and α are the annealing rates of the X hybrid with 5'X'T and 3'X'T, respectively, ε is the conversion rate from X/3'X'T to dsDNA, γ is the productive rate of X by dsDNA, thus,

$\frac{dc_X}{dt} = -\theta c_X c_T$ $\frac{dc_X}{dt} = -\alpha c_X c_T$ $\frac{dc_{X3'X'T}}{dt} = -\varepsilon c_{X3'X'T}$

$\frac{dc_{dsDNA}}{dt} = -\gamma c_{dsDNA}$ $\frac{dc_T}{dt} = -\theta c_X c_T$ $\frac{dc_T}{dt} = -\alpha c_X c_T$

$\frac{dc_{dsDNA}}{dt} = \varepsilon c_{X3'X'T}$ $\frac{dc_X}{dt} = \gamma c_{dsDNA}$ $\frac{dc_{X5'X'T}}{dt} = \theta c_X c_T$

$\frac{dc_{X3'X'T}}{dt} = \alpha c_X c_T$

Therefore,

$$\frac{dc_X}{dt} = \gamma c_{dsDNA} - \theta c_X c_T - \alpha c_X c_T \quad (1)$$

$$\frac{dc_T}{dt} = -\theta c_X c_T - \alpha c_X c_T \quad (2)$$

$$\frac{dc_{X3'X'T}}{dt} = \alpha c_X c_T - \varepsilon c_{X3'X'T} \quad (3)$$

$$\frac{dc_{X5'X'T}}{dt} = \theta c_X c_T \quad (4)$$

$$\frac{dc_{dsDNA}}{dt} = \varepsilon c_{X3'X'T} - \gamma c_{dsDNA} \quad (5)$$

Due to the strand-displacement activity of polymerase, a new dsDNA is formed as soon as a new X is produced in $dsDNA \rightarrow X$. The alteration of c_{dsDNA} can be ignored. Thus,

$$\frac{dc_{dsDNA}}{dt} \approx \varepsilon c_{X3'X'T} \quad (6)$$

The exponential phase occurs early in the amplification reaction before the template becomes exhausted, where $c_T \approx c_{T0}$, but after C_{DS} reaches a steady ratio (λ) with $c_{X3'X'T}$, that is,

$$\frac{c_{dsDNA}}{c_{X3'X'T}} = \lambda \quad (7)$$

$$\frac{\mathrm{d}c_{\mathrm{dsDNA}}}{\mathrm{d}t} = \lambda \frac{\mathrm{d}c_{\mathrm{X3'X'T}}}{\mathrm{d}t} \quad (8)$$

Substitute Eq. (8) into Eq. (6), we can acquire

$$\frac{\mathrm{d}c_{\mathrm{dsDNA}}}{\mathrm{d}t} = \varepsilon c_{\mathrm{X3'X'T}} = \lambda \frac{\mathrm{d}c_{\mathrm{X3'X'T}}}{\mathrm{d}t} \quad (9)$$

Substitute Eq. (3) into Eq. (9), we can obtain

$$\varepsilon c_{\mathrm{X3'X'T}} = \lambda(\alpha c_{\mathrm{X}} c_{\mathrm{T_0}} - \varepsilon c_{\mathrm{X3'X'T}}) \quad (10)$$

The Eq. (10) has a solution of

$$c_{\mathrm{X3'X'T}} = \frac{\lambda}{\varepsilon(\lambda+1)} \alpha c_{\mathrm{X}} c_{\mathrm{T_0}} \quad (11)$$

Substitution of Eq. (11) into Eq. (7), we get

$$c_{\mathrm{dsDNA}} = \lambda c_{\mathrm{X3'X'T}} = \frac{\lambda^2}{\varepsilon(\lambda+1)} \alpha c_{\mathrm{X}} c_{\mathrm{T_0}} \quad (12)$$

Assuming that φ ($1 \geq \varphi \geq 0$) is the ratio of primer that forms extensible primer-template at every round of EXPAR, that is

$$\frac{c_{\mathrm{X3'X'T}}}{c_{\mathrm{X5'X'T}}} = \frac{\varphi}{1-\varphi} \quad (13)$$

$$\frac{\mathrm{d}c_{\mathrm{X3'X'T}}}{\mathrm{d}t} = \frac{\varphi}{1-\varphi} \frac{\mathrm{d}c_{\mathrm{X5'X'T}}}{\mathrm{d}t} \quad (14)$$

Substitute Eq. (14) into Eq. (3), we can obtain

$$\frac{\mathrm{d}c_{\mathrm{X3'X'T}}}{\mathrm{d}t} = \alpha c_{\mathrm{X}} c_{\mathrm{T_0}} - \varepsilon c_{\mathrm{X3'X'T}} = \frac{\varphi}{1-\varphi} \frac{\mathrm{d}c_{\mathrm{X5'X'T}}}{\mathrm{d}t} \quad (15)$$

Substitute Eq. (11) into Eq. (15), we can obtain

$$\frac{\mathrm{d}c_{\mathrm{X3'X'T}}}{\mathrm{d}t} = \frac{1}{(\lambda+1)} \alpha c_{\mathrm{X}} c_{\mathrm{T_0}} = \frac{\varphi}{1-\varphi} \frac{\mathrm{d}c_{\mathrm{X5'X'T}}}{\mathrm{d}t} \quad (16)$$

Substitute Eq. (4) into Eq. (16), we can obtain

$$\frac{\mathrm{d}c_{\mathrm{X3'X'T}}}{\mathrm{d}t} = \frac{1}{(\lambda+1)} \alpha c_{\mathrm{X}} c_{\mathrm{T_0}} = \frac{\varphi}{1-\varphi} \frac{\mathrm{d}c_{\mathrm{X5'X'T}}}{\mathrm{d}t} = \frac{\theta\varphi}{1-\varphi} c_{\mathrm{X}} c_{\mathrm{T_0}} \quad (17)$$

From the Eq. (17), we can obtain

$$\theta = \frac{1-\varphi}{\varphi(\lambda+1)} \alpha \quad (18)$$

Substitution of Eq. (12) and (18) into Eq. (1), a new rate equation can be obtained,

$$\frac{\mathrm{d}c_{\mathrm{X}}}{\mathrm{d}t} = \left[\frac{\gamma\lambda^2}{\varepsilon(\lambda+1)} \alpha c_{\mathrm{T_0}} - \frac{1-\varphi}{\varphi(\lambda+1)} \alpha c_{\mathrm{T_0}} - \alpha c_{\mathrm{T_0}}\right] c_{\mathrm{X}} = \beta c_{\mathrm{X}} \quad (19)$$

Where $\beta = \frac{\gamma\lambda^2}{\varepsilon(\lambda+1)} \alpha c_{\mathrm{T_0}} - \frac{1-\varphi}{\varphi(\lambda+1)} \alpha c_{\mathrm{T_0}} - \alpha c_{\mathrm{T_0}}$

Integration of the Eq. (19), c_{X} can be achieved.

$$\ln c_{\mathrm{X}}\Big|_{c_{\mathrm{X}0}}^{c_{\mathrm{X}}} = (\beta t + \mathrm{A})\Big|_0^t \quad ,$$

$$c_{\mathrm{X}} = \mathrm{e}^{\beta t} c_{\mathrm{X}_0} \tag{20}$$

The Eq. (20) indicates that target oligo is replicated exponentially with an amplification factor (*F*) of $\mathrm{e}^{\beta t}$, and the value of *F* is positively related to *γ* and *φ*.

S3. Amplification fold simulation

Table S2. Outcome at each amplification cycle

Amplification cycle	Released X	Total released X	Total amount of X
1	φX	φX	φX+X
2	$\varphi(\varphi+1)$X	φX+$\varphi(\varphi+1)$X	φX+$\varphi(\varphi+1)$X+X
3	$\varphi(\varphi+1)^2$X	φX+$\varphi(\varphi+1)$X+$\varphi(\varphi+1)^2$X	φX+$\varphi(\varphi+1)$X+$\varphi(\varphi+1)^2$X+X
⋮	⋮	⋮	⋮
n	$\varphi(\varphi+1)^{n-1}$X	$(\varphi+1)^n$X-X	$(\varphi+1)^n$X

S4. Hybridization of primer with strandard or biotin template

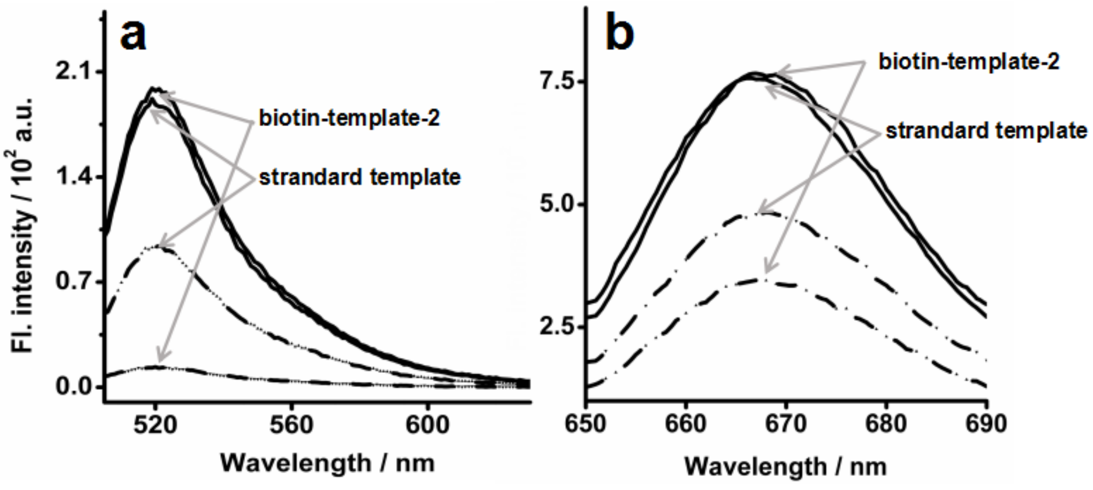

Figure S1. Hybridization of primer with strandard or biotin template. Fluorescence spectra of the FAM/CY5-labelled standard template or biotin-template-2 before (solid lines) and after (dash lines) hybridized with the BHQ1/BHQ3-labelled let-7a under the excitation wavelengths of 490 nm (a) or 640 nm (b).

S5. Ideal and experimental amplification fold

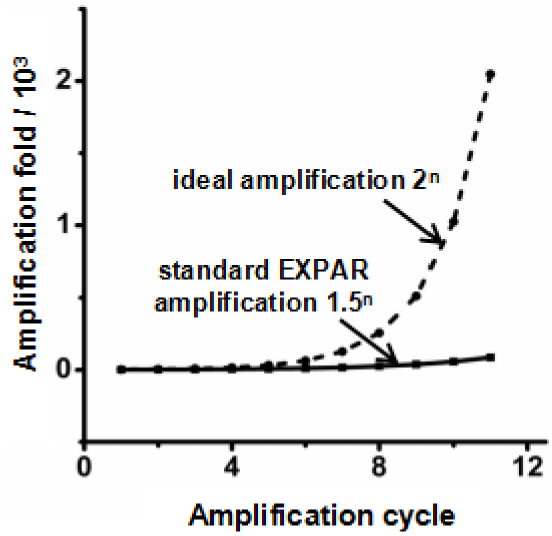

Figure S2. Simulation of the Ideal and experimental amplification fold. Plots for ideal and experimental amplification fold of EXPAR versus amplification cycle.

S6. EXPAR on biotin-template-2,6

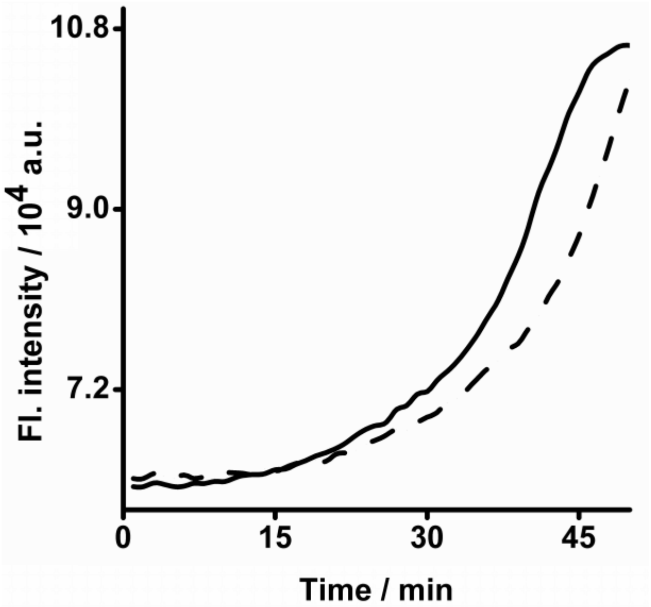

Figure S3. EXPAR on biotin-template-2,6. Real-time fluorescence spectra of EXPAR reactions at 55 °C in response to 10 fmol let-7a (solid line) or without let-7a (dash line). The EXPAR reactions were performed with 0.05 U μL^{-1} Vent (exo-) DNA polymerase, 0.4 U μL^{-1} Nt.BstNBI NEase, and 0.1 μM biotin-template-2,6.

S7. Optimization of the amount of DNA polymerase

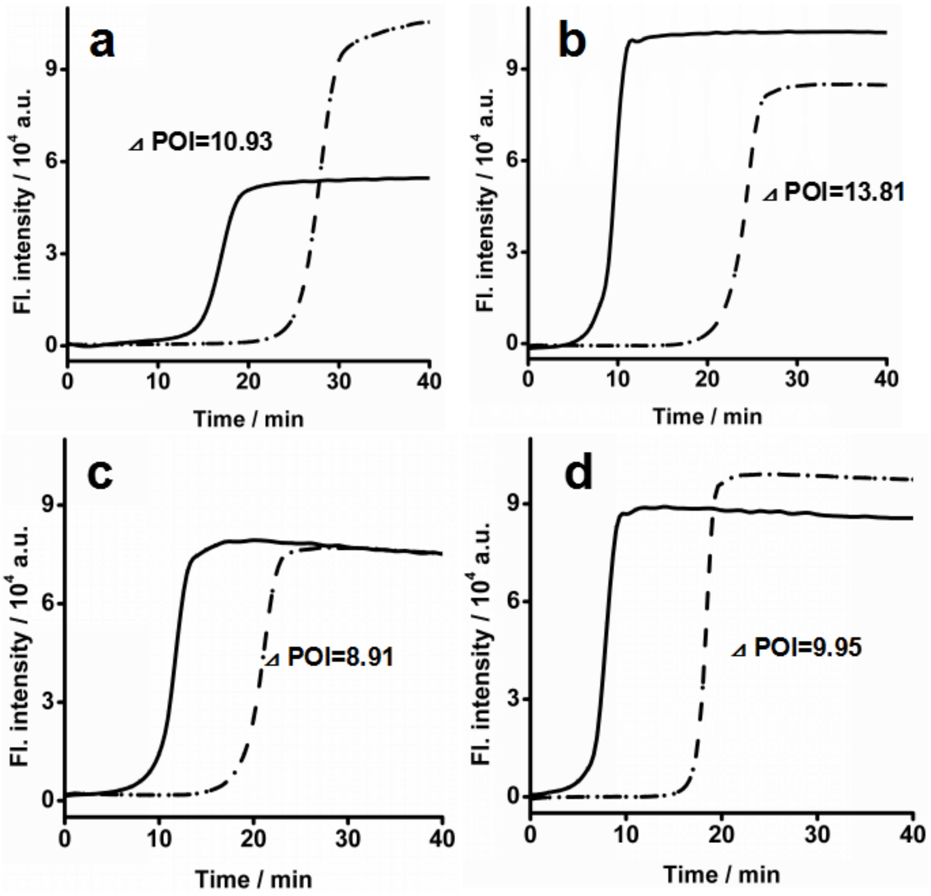

Figure S4. Optimization of the amount of DNA polymerase. Real-time fluorescence spectra of EXPAR reactions at 55 °C in response to 10 fmol let-7a (solid line) or without let-7a (dash line). The EXPAR reactions were performed with 0.4 U μL^{-1} Nt.BstNBI NEase, 0.1 μM biotin-template-2, and (**a**) 0.02, (**b**) 0.05, (**c**) 0.08, and (**d**) 0.10 U μL^{-1} Vent (exo-) DNA polymerase. The maximum interval of the POI between the measurement of 10 fmol let-7a and blank (without let-7a) was found at the Vent (exo-) DNA polymerase of 0.05 U μL^{-1}.

S8. Optimization of the amount of ENase

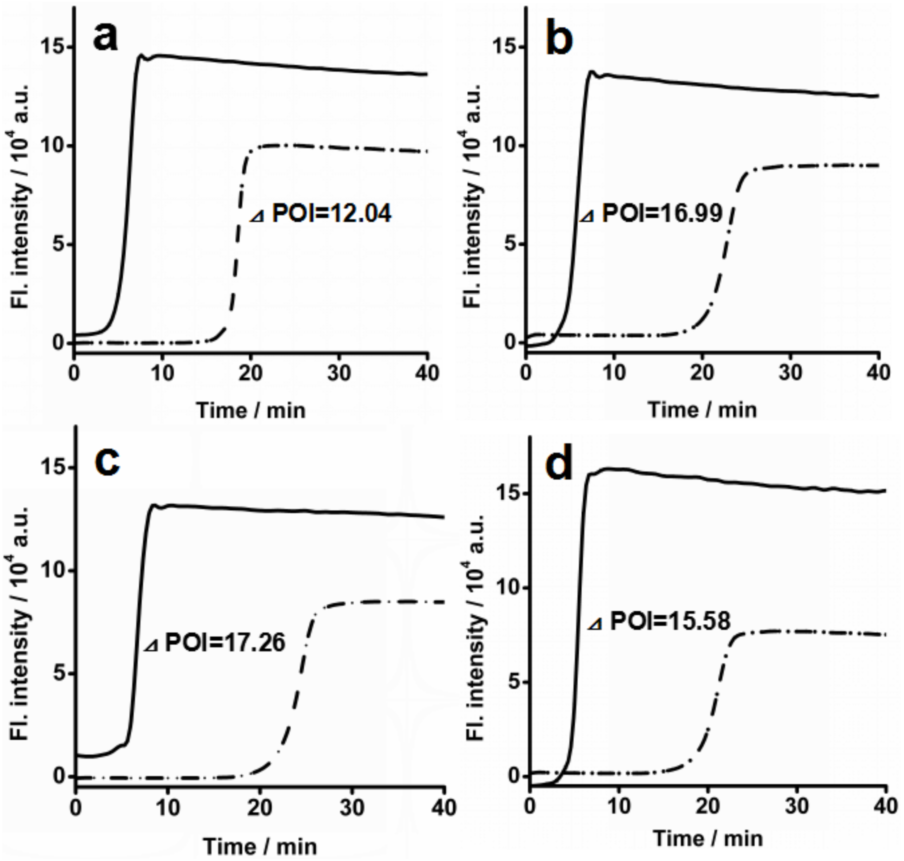

Figure S5. Optimization of the amount of Enase. Real-time fluorescence spectra of EXPAR reactions at 55 ^{o}C in response to 10 fmol let-7a (solid line) or without let-7a (dash line). The EXPAR reactions were performed with 0.05 U μL^{-1} Vent (exo-) DNA polymerase, 0.1 μM biotin-template-2, and (**a**) 0.2, (**b**) 0.3, (**c**) 0.4, and (**d**) 0.5 U μL^{-1} Nt.BstNBI NEase. The maximum interval of the POI between the measurement of 10 fmol let-7a and blank (without let-7a) was found at the Nt.BstNBI NEase of 0.4 U μL^{-1}.

S9. Optimization of the amount of template

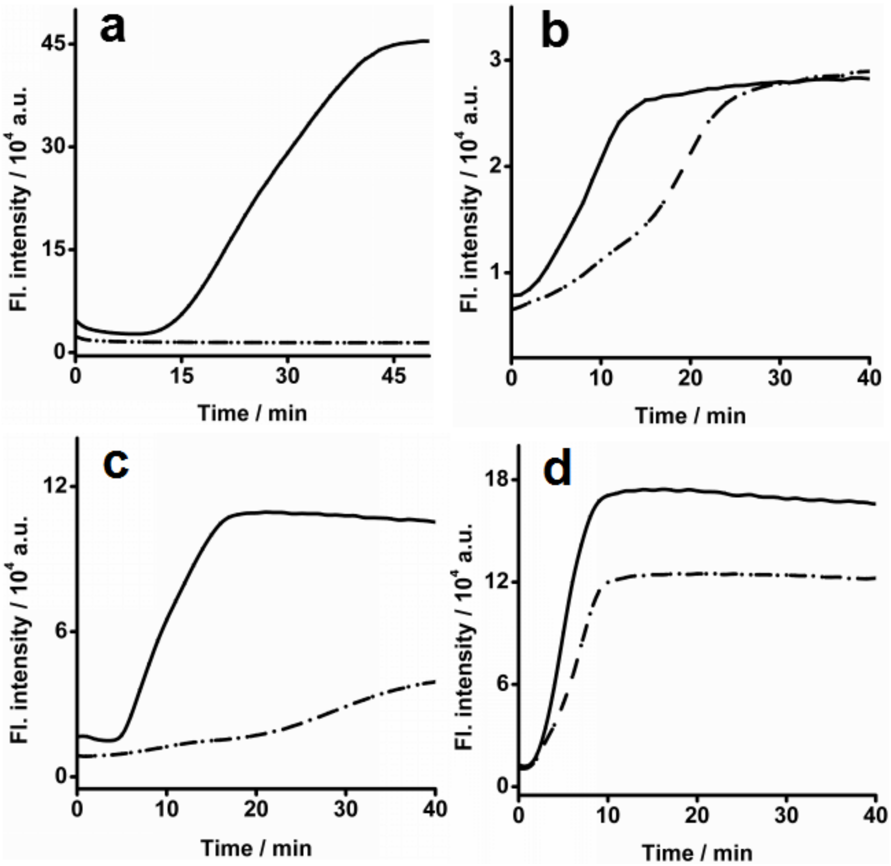

Figure S6. Optimization of the amount of template. Real-time fluorescence spectra of EXPAR reactions at 55 °C in response to 10 fmol let-7a (solid line) or without let-7a (dash line). The EXPAR reactions were performed with 0.05 U μL^{-1} Vent (exo-) DNA polymerase, 0.4 U μL^{-1} Nt.BstNBI NEase, and (**a**) 0.02, (**b**) 0.05, (**c**) 0.10, and (**d**) 0.20 μM biotin-template-2. The utility of 0.02 μM bioten-template-2 gives the maximum interval of the POI between the measurement of 10 fmol let-7a and blank (without let-7a), however, the amplification time is too long. In concendiation of the reaction speed and sensitivity, the optimal template amount was chosen as 0.10 μM.

S10. Optimization of reaction temperature

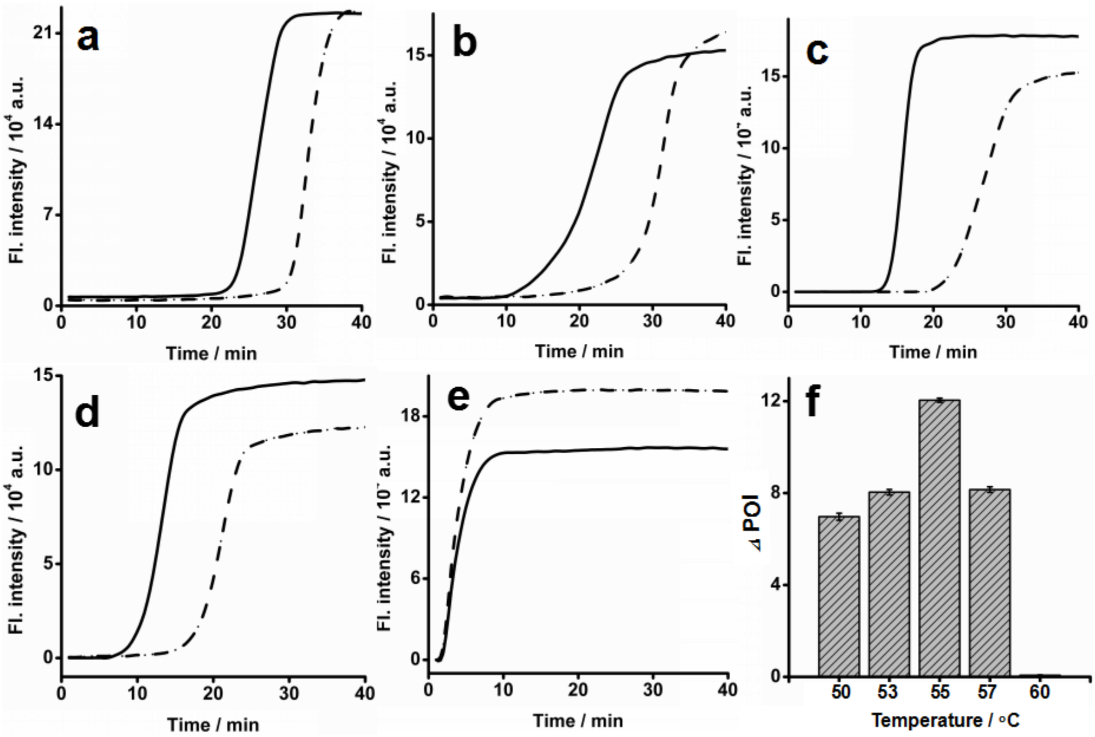

Figure S7. Optimization of reaction temperature. Real-time fluorescence spectra of EXPAR reactions in response to 10 fmol let-7a (solid line) or without let-7a (dash line). The EXPAR reactions were performed with 0.05 U μL^{-1} Vent (exo-) DNA polymerase, 0.4 U μL^{-1} Nt.BstNBI NEase, and 0.10 μM biotin-template-2 at (**a**) 50 ^{o}C, (**b**) 53 ^{o}C, (**c**) 55 ^{o}C, (**d**) 57 ^{o}C and (**e**) 60 ^{o}C. (**f**) Histogram of ΔPOI with temperature. The maximum interval of the POI between the measurement of 10 fmol let-7a and blank (without let-7a) was found at 55 ^{o}C.

S11. Estimation of interference of EXPAR

The interferences for the detection of let-7a arisen from its family members were evaluated as follows. Assuming the correlation equation for let-7a determination is $\mathrm{POI} = \mathrm{b} + \mathrm{a}\lg A_{\mathrm{miRNA}}$. Therefore, $\mathrm{POI}_{\mathrm{let-7x}} - \mathrm{POI}_{\mathrm{let-7a}} = \mathrm{a}(\lg A_{\mathrm{let-7x}} - \lg A_{\mathrm{let-7a}})$, $\lg\frac{A_{\mathrm{let-7x}}}{A_{\mathrm{let-7a}}} = \frac{\mathrm{POI}_{\mathrm{let-7x}} - \mathrm{POI}_{\mathrm{let-7a}}}{\mathrm{a}}$. The interference (I) = $10^{\frac{POI_{let-7x}-POI_{let-7a}}{a}}$.

Table S3. Evaluation of the interference for biotin-template based EXPAR

	Let-7a	Let-7b	Let-7c	Let-7d	Let-7e	Let-7f	Let-7g	Let-7i
POI	12.6	25.75	23.71	19.07	14.47	20.32	21.28	26.11
I	—	1.5×10^{-5}	1.4×10^{-4}	4.4×10^{-2}	9.33	1.1×10^{-2}	2.3×10^{-3}	1.0×10^{-5}

Table S4. Evaluation of the interference for standard EXPAR

	Let-7a	Let-7b	Let-7c	Let-7d	Let-7e	Let-7f	Let-7g	Let-7i
POI	24.06	31.62	28.61	26.14	25.22	26.68	27.79	32.13
I	—	1.9×10^{-4}	3.6×10^{-2}	2.7	13.2	1.04	0.15	7.8×10^{-5}

Table S5. Evaluation of the interference for toehold/biotin-template-based EXPAR

	Let-7a	Let-7b	Let-7c	Let-7d	Let-7e	Let-7f	Let-7g	Let-7i
POI	14.05	27.12	25.79	23.02	21.86	24.32	24.94	27.53
I	—	2.7×10^{-5}	1.2×10^{-4}	3.2×10^{-3}	1.2×10^{-2}	6.9×10^{-4}	3.4×10^{-4}	1.7×10^{-5}

S12. Melting temperature investigation of toehold/biotin-template-2

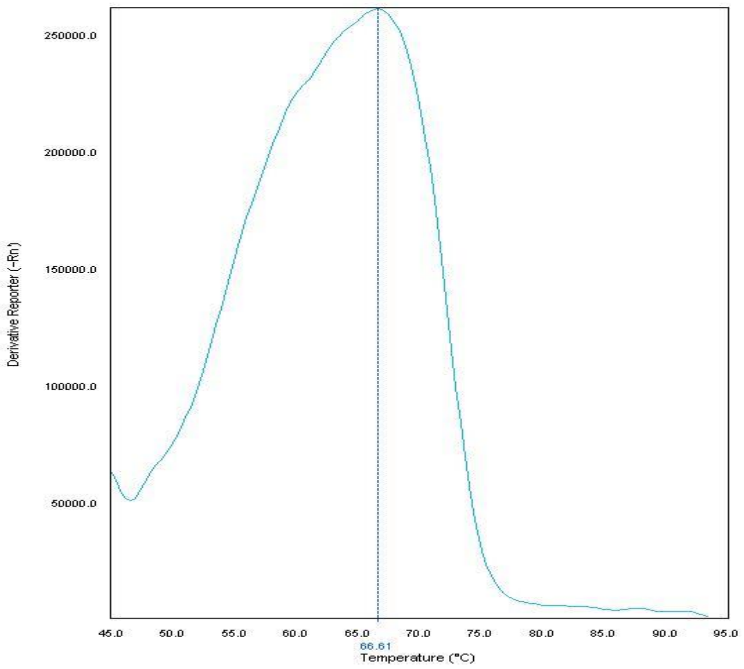

Figure S8. Melting temperature investigation of toehold/biotin-template-2. Tm curves were obtained from the derivative of fluorescence intensity signal as the function of temperature.

S13. Table S6. Comparison of the analytical performance of different methods[a]

Methods	Amplification mode	Detection limit	Dynamic range （orders of magnitude）	reference
Microarray	_	1 fmol	2	1
Northern blotting	_	1 fmol	3	2
Bioluminescence	_	1 fmol	5	3
competitive binding assay	_	228 amol	5	4
Electrochemistry	_	200 amol	4	5
DSNSA	Linear	80 amol	4	6
Branched RCA	Linear	6 amol	3	7

SPRI	Linear	5 amol	4	8
Hairpin-based EXPAR	Exponential	3.8 amol	4	9
LCR	Linear	3.5 amol	3	10
SDA	Linear	2.1 amol	4	11
LAMP	Exponential	1 amol	6	12
CPB	Linear	1 amol	1	13
GO-based EXPAR	Exponential	540 zmol	3	14
Exponential SDA	Exponential	16 zmol	9	15
P-ERCA	Exponential	0.24 zmol	3	16
EXPAR	Exponential	0.1 zmol	10	17
HQEA	Quadratic	0.05 zmol	5	18
Biotin-based EXPAR	Exponential	0.001zmol	12	This work
Toehold/biotin-based EXPAR	Exponential	0.01zmol	10	This work

[a]Summary of homogeneous binding assays for the detection of nucleic acid with detection scheme of fluorescent. DSNSA: duplex-specific nuclease signal amplification; RCA: rolling circle amplification; SPRI: Surface plasmon resonance imaging; Hairpin-based EXPAR: Hairpin Probe-Based Circular Exponential Amplification; LCR: ligase chain reaction; SDA: strand-displacement amplification; LAMP: loop-mediated isothermal amplification; CPB: conjugated-polymer-based methods; GO-based EXPAR: graphene oxide (GO) fluorescence switch-based circular exponential amplification; Exponential-SDA: Exponential Strand-Displacement Amplification; P-ERCA: padlock probe-based exponential rolling circle amplification; EXPAR: exponential amplification reaction; HQEA: hairpin-mediated quadratic enzymatic amplification

REFERENCES

1. Lee, J. M., Cho, H. and Jung, Y. (2010) Fabrication of a structure-specific RNA binder for

array detection of label-free microRNA. *Angew. Chem. Int. Ed.,* **49**, 8662–8665.

2. Válóczi, A., Hornyik, C., Varga, N., Burgyán, J., Kauppinen,S. and Havelda, Z. (2004) Sensitive and specific detection of microRNAs by northern blot analysis using LNA-modified oligonucleotide probes. *Nucleic Acids Res.*, **32**, e175.
3. Cissell, K. A., Rahimi, Y., Shrestha, S., Hunt, E. A. and Deo, S. K. (2008) Bioluminescence-based detection of microRNA, miR21 in breast cancer cells. *Anal. Chem.,* **80**, 2319–2325.
4. Wang, W., Kong, T., Zhang, D., Zhang, J. A. and Cheng, G. S. (2015) Label-free microRNA detection based on fluorescence quenching of gold nanoparticles with a competitive hybridization. *Anal. Chem.,* **87**, 10822–10829.
5. Gao, Z. and Yang, Z. (2006) Detection of microRNAs using electrocatalytic nanoparticle tags. *Anal. Chem.,* **78**, 1470–1477.
6. Lin, X., Zhang, C., Huang, Y., Zhu, Z., Chen, X. and Yang, C. J. (2013) Backbone-modified molecular beacons for highly sensitive and selective detection of microRNAs based on duplex specific nuclease signal amplification. *Chem. Commun.,* **49**, 7243–7245.
7. Cheng, Y. Q., Zhang, X., Li, Z. P., Jiao, X., Wang, Y. and Zhang, Y. (2009) Highly sensitive determination of microRNA using target-primed and branched rolling-circle amplification. *Angew. Chem. Int. Ed.*, **48**, 3268–3272.
8. Fang, S., Lee, H. J., Wark, A. W. and Corn, R. M. (2006) Attomole microarray detection of microRNAs by nanoparticle-amplified SPR imaging measurements of surface polyadenylation reactions. *J. Am. Chem. Soc.,* **128**, 14044–14046.
9. Wang, G. L. and Zhang, C. Y. (2012) Sensitive detection of microRNAs with hairpin probe-based circular exponential amplification assay. *Anal. Chem.*, **84**, 7037–7042.
10. Yan, J. L., Li, Z. P., Liu, C. H. and Cheng, Y. Q. (2010) Simple and sensitive detection of microRNAs with ligase chain reaction. *Chem. Commun.,* 2010, **46**, 2432–2434.
11. Dong, H., Zhang, J., Ju, H., Lu, H., Wang, S., Jin, S., Hao, K., Du, H. and Zhang, X. (2012) Highly sensitive multiple microRNA detection based on fluorescence quenching of graphene oxide and isothermal strand-displacement polymerase reaction. *Anal. Chem.* 2012, **84**, 4587–4593.

12. Li, C. P., Li, Z. P., Jia, H. X. and Yan, J. L. (2011) One-step ultrasensitive detection of microRNAs with loop-mediated isothermal amplification (LAMP). *Chem. Commun.,* 2011, **47**, 2595–2597.

13. Fan, Y., Chen, X. T., Trigg, A. D., Tung, C., Kong, J. M. and Gao, Z. Q. (2007) Detection of microRNAs using target-guided formation of conducting polymer nanowires in nanogaps. *J. Am. Chem. Soc.,* **129**, 5437–5443.

14. Liu, H. Y., Li, L., Wang, Q., Duan, L. L. and Tang, B. (2014) Graphene fluorescence switch-based cooperative amplification: a sensitive and accurate method to detection microRNA. *Anal. Chem.*, **86**, 5487–5493.

53. Shi, C., Liu, Q., Ma, C. P. and Zhong, W. W. (2014) Exponential strand-displacement amplification for detection of microRNAs. *Anal. Chem.,* **86**, 336–339.

16. Liu, H. Y., Li, L., Duan, L. L., Wang, X., Xie, Y. X., Tong, L. L., Wang, Q. and Tang, B. (2013) High specific and ultrasensitive isothermal detection of microRNA by padlock probe-based exponential rolling circle amplification. *Anal. Chem.*, **85**, 7941–7947.

17. Jia, H., Li, Z. P., Liu, C. and Cheng, Y. Q. (2010) Ultrasensitive detection of microRNAs by exponential isothermal amplification. *Angew. Chem. Int. Ed.*, **49**, 5498–5501.

18. Duan, R. X., Zuo, X. L., Wang, S. T., Quan, X. Y., Chen, D. L., Chen, Z. F., Jiang, L., Fan, C. H. and Xia, F. (2013) Lab in a tube: ultrasensitive detection of microRNAs at the single-cell level and in breast cancer patients using quadratic isothermal amplification. *J. Am. Chem. Soc.,* **135**, 4604–4607.
